# Impact of Nitrogen Nutrition on *Cannabis sativa*: An Update on the Current Knowledge and Future Prospects

**DOI:** 10.3390/ijms20225803

**Published:** 2019-11-18

**Authors:** Simone Landi, Roberto Berni, Giorgia Capasso, Jean-Francois Hausman, Gea Guerriero, Sergio Esposito

**Affiliations:** 1 Department of Biology, Complesso Universitario di Monte Sant’Angelo, University of Naples “Federico II”, Via Cinthia, I-80126 Napoli, Italy; simone.landi@unina.it (S.L.); giorgia.271291@gmail.com (G.C.); 2 Department of Life Sciences, University of Siena, via P.A. Mattioli 4, I-53100 Siena, Italy; berni10@student.unisi.it; 3 Trees and Timber Institute-National Research Council of Italy (CNR-IVALSA), via Aurelia 49, 58022 Follonica (GR), Italy; 4 Environmental Research and Innovation Department, Luxembourg Institute of Science and Technology, 5, rue Bommel, Z.A.E. Robert Steichen, L-4940 Hautcharage, Luxembourg; jean-francois.hausman@list.lu

**Keywords:** *Cannabis sativa*, drought, salinity, nitrogen, bast fibers, secondary metabolism, next-generation sequencing

## Abstract

Nitrogen (N) availability represents one of the most critical factors affecting cultivated crops. N is indeed a crucial macronutrient influencing major aspects, from plant development to productivity and final yield of lignocellulosic biomass, as well as content of bioactive molecules. N metabolism is fundamental as it is at the crossroad between primary and secondary metabolic pathways: Besides affecting the synthesis of fundamental macromolecules, such as nucleic acids and proteins, N is needed for other types of molecules intervening in the response to exogenous stresses, e.g. alkaloids and glucosinolates. By partaking in the synthesis of phenylalanine, N also directly impacts a central plant metabolic ‘hub’—the phenylpropanoid pathway—from which important classes of molecules are formed, notably monolignols, flavonoids and other types of polyphenols. In this review, an updated analysis is provided on the impact that N has on the multipurpose crop hemp (*Cannabis sativa* L.) due to its renewed interest as a multipurpose crop able to satisfy the needs of a bioeconomy. The hemp stalk provides both woody and cellulosic fibers used in construction and for biocomposites; different organs (leaves/flowers/roots) are sources of added-value secondary metabolites, namely cannabinoids, terpenes, flavonoids, and lignanamides. We survey the available literature data on the impact of N in hemp and highlight the importance of studying those genes responding to both N nutrition and abiotic stresses. Available hemp transcriptomic datasets obtained on plants subjected to salt and drought are here analyzed using Gene Ontology (GO) categories related to N metabolism. The ultimate goal is to shed light on interesting candidate genes that can be further studied in hemp varieties growing under different N feeding conditions and showing high biomass yield and secondary metabolite production, even under salinity and drought.

## 1. Introduction

In recent years, hemp (*Cannabis sativa* L.) has been the object of research due to its potential as crop with multiple uses in a required bio-safe agriculture and economy [1,2,3]. Hemp is one of the earliest domesticated crops [4] and is commonly used in over 25,000 commercial products [5]. Industrial hemp differs from psychotropic varieties in the tetrahydrocannabinol (THC) content which is <0.3% and it is cultivated both for seed oil and fibers [6]. The cortex of the hemp stalk contains highly crystalline cellulosic fibers, known as bast fibers, which mechanically support the phloem. These fibers are strong and long and find application as green substitutes of synthetic fibers in biocomposites.

**Figure 1:**
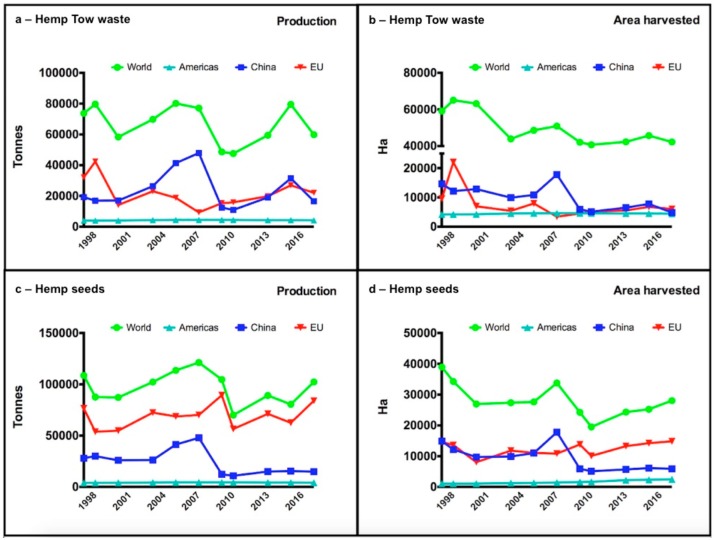
Production (**a**,**c**) and area harvested (**b**,**d**) of hemp (tow waste and seeds) from 1997 to 2017. The figure was obtained from data present in the FAO database (www.fao.org/stats). FAOSTAT reports no data for Canada in the selected period.

Hemp is polyvalent in terms of applications, as it also produces interesting secondary metabolites: The industrial varieties contain cannabidiol (CBD) [7], but also other interesting compounds from a pharmaceutical point of view, such as specific terpenes, responsible for the typical scent. Considering these medical and industrial applications, nowadays hemp is extensively cultivated in almost 50 countries [2,3,8,9]. Interestingly, among the 16 best producers, 11 are European countries (e.g., France, Austria, Italy, among others) [3]. The production of hemp tow waste and seeds constantly increased worldwide from 1997–2007; then a reduction was observed until 2010. In the last decade, an increase was again recorded, indicating European Union (EU) countries as the best producers both for hemp seeds and—in the recent years— tow waste, overtaking China (Figure 1).

Data about harvested area reflect the production: American countries showed a lower production, resulting in USA as the major hemp importer worldwide [5].

This reignited the interest of USA in hemp cultivation, obviously induced by an increased market demand. The major hemp products, namely fiber and CBD, showed market prices ranging from 4848 $/Ha of fiber to the 25000 $/Ha of CDB [3]. This directed the scientific research to improve the knowledge about stem development, genetic regulation of fiber traits, secondary metabolites, biosynthetic pathways and their engineering, increase of grain yield, oil composition, and response to biotic and abiotic stresses [3].

The availability of N affects the response of plants to exogenous cues; therefore, understanding the regulation of N metabolism under stress is crucial for agriculture. N represents indeed a key macronutrient for plant cells and its availability influences major aspects of plant physiology, such as photosynthesis, development, growth, flowering, and senescence [10,11].

Nevertheless, despite the agro-economic interest for hemp, many aspects affecting the productivity of this crop remain still poorly studied. N availability is an example: Its impact on hemp growing under abiotic stress conditions is even less understood [3]. Besides the impact on plant biomass accumulation, mineral nutrition can also affect the production of secondary metabolites, as for example shown for the cannabinoid metabolism [12].

Hemp shows a number of interesting agricultural features, such as low nitrogen (N) input requirement [2,13,14,15], heat tolerance [2,15], phytoremediation [16,17], positive effects on environments [18], benefits in crop rotation [19] and availability of high-yield varieties [20,21].

**Figure 2:**
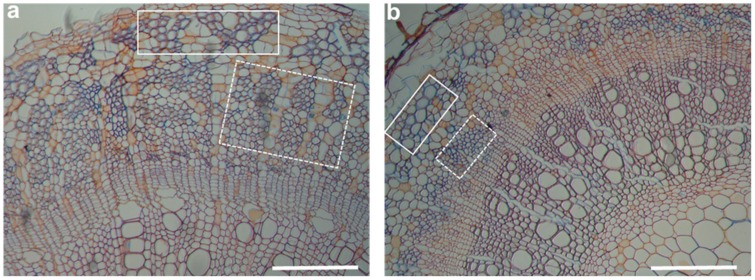
Cross sections of hemp hypocotyls aged 4 weeks and grown in the absence (**a**) and presence (**b**) of NaCl 200 mM. The white boxes show primary bast fibers, while the dotted ones indicate secondary bast fibers. Scale bars: 200 µm.

Hemp production is particularly threatened by abiotic stresses and N starvation, the major constraints limiting crop yield [2,21,22]; it must be underlined that these two factors are strictly correlated, severely impacting cell metabolism, plant growth and differentiation [23]. Abiotic stresses induce a number of morphological, physiological and molecular changes affecting plant growth and productivity [10]; this is particularly true in hemp, where photosynthesis-related pathways and associated genes are strongly downregulated [24]. Drought and salinity seriously threaten agricultural productivity. Important effects are observed in the stem tissues of salt-stressed hemp: Besides the presence of smaller xylem vessels [25], a decrease in the number of bast fibers is observed (Figure 2). This is particularly evident for secondary bast fibers (Figure 2, dotted box). Environmental stress significantly affects two important feature of hemp commercial varieties, i.e., stem and seed yield. The relationship between yield and environmental constraints was due to flowering time, mainly regulated by photoperiod and temperature [15].

Humic acids together with macronutrients—such as N, K, and P—were shown to affect the cannabinoid profile; this effect is depending on plant organs and acts in a spatial-dependent manner (e.g., top-middle-bottom of the plant) [12]: For example, humic acids reduced the variability in cannabinoid abundance in the different organs (flowers, leaves, inflorescences), but this increased homogeneity was accompanied by a decrease in the upper regions of plants, which normally contain high levels of these secondary metabolites.

In light of the central role of N as macronutrient affecting both primary and secondary plant metabolic pathways, we here provide an overview of the current knowledge on hemp physiology in relation with N nutrition. We also report genes related to N metabolism and responding to salt/drought stress in hemp to pave the way to future strategies improving specific traits under unfavorable conditions for this economically important multipurpose crop.

## 2. Physiological Effects of N Availability in Hemp

The availability of macronutrients in the soil heavily affects plant growth and development [10,11]. When soils are depleted of particular nutrients, severe limitations in biomass production occur [11]. These effects are particularly true when N nutrition is considered, due to the essential role of N in the structure of crucial molecules for life, such as amino acids and N-bases. Therefore, it is not unexpected that plants evolved a number of mechanisms to avoid severe damage when N is limiting in the soil [11]. On the other hand, it is not rare that plants can experience a prolonged limitation of N; when this occurs, recycling mechanisms have been developed to avoid—at least in part—growth stop and/or limiting stress symptoms, in order to guarantee plant survival [26]. Nutrient starvation may cause an important physiological process, nutrient resorption; this parameter contributes to nutrient retention and it is intended as a strategy for nutrient storage [26,27]. Nutrient resorption is defined as the percentage of a nutrient stored by a plant before the beginning of the senescence process (physiological or stress-induced) and the resorption proficiency as the final concentration of a specific nutrient in tissues after senescence [26]. Resorption process allows the recycling primarily of N and P and it is intuitive that plants living in nutrient-limiting soils or in stress environments (e.g. arid ecosystems) show better resorption capabilities [27]. This aspect was investigated in hemp in comparison with other important species in semi-arid environments ([28], summarized in Table 1). Hemp yield is limited by nutrient availability and this is particularly true in semi-arid ecosystems, where drought conditions are often accompanied by N deficiency [2,21]. Interestingly, N resorption efficiency (NRE) showed a general decrease in *Cannabis* plants in response to increased soil N availability. Under control conditions, hemp showed the best NRE value as compared to *Artemisia scoparia*, *Chenopodium acuminatum* and *Phragmites communis*. Furthermore, hemp showed a high NRE in the absence of N and upon N- and phosphorus (P)-enriched environments. Less difference was reported between the analyzed species for PRE (phosphorus resorption efficiency) [28].

The effects of N supply on photosynthetic N-use efficiency of plant canopy (PNUEc) are determined by the effect of N on the size of canopy and/or leaf area index (LAI). PNUEc of hemp increased with decreasing N fertilization and this is correlated with a reduction in LAI [2]. Furthermore, these effects could be correlated to a variation in the absolute amount of the specific leaf N content (SLN). Interestingly, upon water scarcity, hemp showed a concomitant decrease of PNUEc value and increase of canopy photosynthetic water use efficiency (PWUEc). This parameter showed no or less differences under varying N inputs [2].

Experiments carried out in Eastern Canada (Québec) revealed the effects of N, P, and K fertilization on the biomass and seed yield in two hemp cultivars, CRS-1 and Anka [29]. The results revealed the existence of a strong interaction environment x fertilization, as well as a dependence on the cultivar. A more than two-fold seed yield was obtained with 200 kg N/ha; an effect was observed on cellulose and hemicellulose content as well, but it was minimal. The final recommendation was to use N fertilization >200 kg N/ha, which is higher than the level normally used in Western Canada, i.e., 150 kg N/ha [29]. This study indicates the importance of evaluating the geographical location and relative environmental conditions (e.g., Eastern Canada is more humid than Western Canada) before establishing agronomic recommendations relative to N fertilization of hemp cultures.

Another study evaluated the impact of N fertilization on photosynthesis, fibers and seed oil content in a cultivar of hemp grown in Latvia [30]. High doses of N (100 kg/ha and in the form of NH_4_NO_3_) increased the content of chlorophyll already 7 days after the first application and improved the Performance Index (PI) by increasing photosystem II activity. Although the height of plants increased by 11% with N, the fiber yield was ca. 8% lower than non-fertilized plants: this indicates a carbon-dependent assimilation of nitrate into amino acids which lowers carbohydrate biosynthesis. High N can indeed affect the mechanical strength of stems and increase lodging by reducing both cellulose and lignin contents [31]. The composition of seed oil did not change significantly upon different fertilizations, although a general decrease was observed, as seen with fibers, a finding suggesting a preferential metabolic shunt towards amino acid and protein biosynthesis.

It is worth mentioning here the beneficial effect of plant growth promoting bacteria (PGPB) on growth and development: PGPB indeed improve crop yield by enhancing nutrient mobilization and protecting against exogenous stresses [32]. Beneficial bacteria greatly increase the root surface area, thereby improving nutrients’ uptake from the soil; some bacteria can fix N, thus improving the fitness of plants in N-limited environments; other micro-organisms are able to solubilize P salts and thus allow access to otherwise recalcitrant forms [33]. A recent perspective article discussed the interest of testing PGPB on hemp [34] as biostimulants; it was shown that PGPB clearly improve nutrient use, tolerance to (a)biotic stresses, and accumulation of phytochemicals. Treatment of *C. sativa* with the biostimulant Mammoth P™ improved growth, but a higher amount of cannabinoids could not be detected [35].

## 3. N Nutrition and Impact on the Plant Secondary Metabolism

N is well known as the primary element taken up from soil and it is necessary to plants for primary growth; on the other hand, N is an essential component in many plant secondary metabolites; therefore, its availability in soils affects not only biomass production, but the synthesis and final yield of specific molecules as well.

An emblematic example is represented by alkaloids: In poppy (*Papaver somniferum* L.), N split supplementation at the stages of leaf rosette and flowering increased alkaloid yield of capsules. Morphine was found at the highest levels in plants after the treatment with the highest dose of N (supplied as NH_4_NO_3_) [36].

Alkaloids can be synthesized from polyamines, nitrogenous compounds responding to N nutrition and involved in plants’ defense responses by contributing to stiffen the cell wall via the H_2_O_2_ released by amine oxidases, or stimulating the synthesis of secondary metabolites via the action of the products deriving from their oxidation [37]. Hemp contains the polyamine-derived alkaloids cannabisativine and anhydrocannabisativine [38]: Intriguingly, a detailed study on their bioactivity and biosynthetic regulation is, to the best of our knowledge, still missing. Therefore, it will be of interest to assess the content of polyamine-derived alkaloids in hemp plants grown under different N feeding conditions to see whether a specific treatment induces the accumulation of alkaloids.

**Figure 3:**
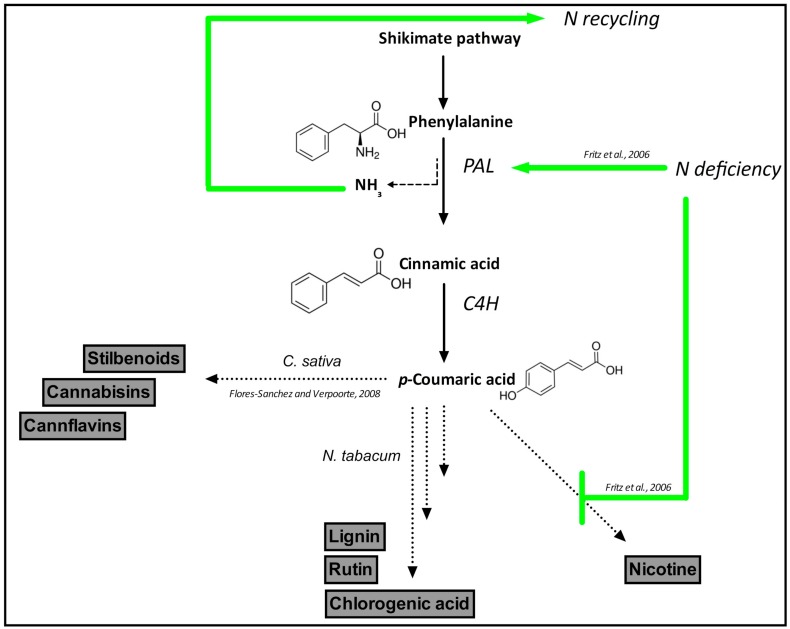
Schematic representation of N role in secondary metabolism in hemp. Tobacco’s pathways are shown for comparative purposes. Abbreviations: PAL = Phenylalanine ammonia lyase; C4H = Cinnamate-4-hydroxylase.

The relationship between alkaloids and phenylpropanoid biosynthesis is particularly interesting when N supplementation is considered: In tobacco, N deficiency causes a metabolic shift from the alkaloid nicotine to carbon-rich phenylpropanoids, with a concomitant increase in lignin, chlorogenic acids and rutin [39] (Figure 2). This finding is interesting if one considers that the ammonia released by phenylalanine ammonia lyase (PAL) is recycled back to fuel an alternative N cycle in plants [40]. Therefore, the stimulation of the phenylpropanoid pathway under N deficiency may represent a mechanism to ensure a basal N cycling under conditions of N scarcity. Studies on the effect of N supplementation in hemp in relation to the production of alkaloids or phenolic compounds are missing, but would be interesting to perform in order to know the effect of specific N feeding conditions on the pathways leading to the synthesis of alkaloids and phenolics (Figure 3).

## 4. Transcriptomic Datasets Identify Genes Involved in the Regulation of N Metabolism and Responsive to Abiotic Stresses in *C. sativa*

Nowadays, next-generation sequencing (NGS) generates a great number of datasets, which can help shed light on the relationship between different metabolic pathways in numerous plant species. Such data are valuable, as they provide a first indication of candidates linking different pathways and therefore interesting for functional studies. Co-clustering of genes (for example sharing the same expression pattern in a given experimental condition) involved in different metabolic pathways indicates the existence of a potential co-regulation. Such results contribute to “feed” useful databases, such as STRING [41] (available at https://string-db.org); these data greatly help in functional findings in high-throughput *-omics* datasets.

To get an overview of salt- and drought-responsive genes of hemp involved in N metabolism, we mined two different NGS datasets [9,24] using N-related GO and KEGG categories. We took advantage of the availability of the sequenced hemp genome to annotate some identified genes in order to compare them with orthologs from other sequenced species [8].

As reported by Gao et al. [24], drought stress induced a total of 1258 differentially expressed genes (DEGs) in hemp, including 394 upregulated and 864 downregulated transcripts. Among these, a significant number of genes belonging to GO categories related to N metabolism were found. In the up- and downregulated categories, 18 and 22 genes were identified, respectively (Table 2). The 18 genes which are upregulated and related to N-metabolism belong to 8 different GO groups: “*response to organic nitrogen*” (GO:0010243); “*nitrogen compound metabolic process genes*” (GO:0006807); “*cellular nitrogen compound biosynthetic process”* (GO:0044271); “*cellular nitrogen compound metabolic process*” (GO:0034641); “*nitrogen compound transport*” (GO:0071705); “*cellular response to nitrogen starvation”* (GO:0006995); “*cellular nitrogen compound catabolic process”* (GO:0044270); “*regulation of nitrogen compound metabolic process*” (GO:0051171).

Interestingly, upregulated genes involved in both drought stress and N metabolism include the gene encoding δ-1-pyrroline-5-carboxylate synthetase (P5CS), the regulatory enzyme of the proline biosynthetic pathway [42], which is well-known to play a central role in drought tolerance in a wide-range of crops [43,44]. Breeding studies addressed to regulate proline accumulation were performed in different plants [22]. Comparison in barley-improved lines and cultivars revealed a key role for P5CS genes as focus for breeding strategies [45]. Similar approaches could be used to obtain drought-resistant hemp cultivars with improved proline accumulation.

A number of transcription factors (TFs) involved in abiotic stress response were upregulated in the drought response dataset (MYB, WRKY, and LHY), which are known to have a role in mineral nutrition. Notably, the role of both MYB and WRKY in nutrient assimilation (e.g., N and P) has been characterized in different crops. MYB TFs such as *At*Phr2, *At*Nsr1, *Lj*MYB101 and *Lj*MYB102 showed the ability to counteract starvation by N and P, inducing tolerance by increasing the expression of N and P transporters, as well as flavonoid biosynthetic genes [46,47]. Similar effects were reported for *Os*WRKY74, a TF responding to the lack of N, P and iron (Fe), as well as to abiotic stresses [48]. Differences in the expression of various TFs, especially MYBs, were reported in fiber-type *vs.* seed-type cultivars of hemp, thereby suggesting a different transcriptional regulation in varieties grown for different industrial purposes [9]. Thus, breeding strategies focused on MYB or WRKY transcription factors could be useful to improve hemp varieties. In support of this, it is worth citing that in *Poaceae Os*MYB55, *Ta*MYB31, *Ta*MYB74, *Ta*WRKY1 and *Ta*WRKY33 were used to obtain modified plants and/or improved varieties showing a better adaptation to adverse environments [44,49,50,51].

Interestingly, abiotic stresses and N levels (starvation/availability) showed contrasting relationships, depending on the species [23,52,53,54,55]. Durum wheat plants subjected to N starvation showed an increased expression of MYB and WRKY transcription factors, as well as aldehyde dehydrogenase [11]. Particularly, the aldehyde dehydrogenase gene family was strictly correlated with N levels and source. This plays critical roles in glycolysis/gluconeogenesis, ascorbate, pyruvate, and propanoate metabolism. Wheat plants subjected to different N regimes showed upregulation of aldehyde dehydrogenase upon high concentration of ammonium and nitrate, while they showed downregulation upon N-free conditions [54].

The 22 downregulated genes in the hemp dataset were subdivided into eight groups: *“nitrogen compound transport genes”* (GO:0071705); “*cellular response to nitrogen starvation*” (GO:0006995); “*nitrogen compound metabolic process*” (GO:0006807); “*nitrogen fixation genes”* (GO:0009399); “*cellular nitrogen compound metabolic process genes*” (GO:0034641); “*nitrogen compound transport*” (GO:0071705); “*detoxification of nitrogen compound gene*” (GO:0051410); “*cellular nitrogen compound biosynthetic process gene* (GO:0044271). Interestingly, downregulated genes involved in both drought and N metabolism include transporters, such as a vacuolar Fe transporter, major facilitator proteins and nitrate carriers. Nitrate carriers and major facilitator proteins were identified as orthologs of the *Arabidopsis thaliana* nitrate transporters *At*NRT1.2 (At1g69850) and *At*NTR1.11 (At1g52190), respectively. These genes play a pivotal role in stress responses and nutritional starvation. *At*NRT1.2 is a root-localized transporter, which also controls ABA transport and biosynthesis, regulating stomata opening [56]; *At*NTR1.11 has been reported as an important low affinity nitrate transporter involved in N redistribution in plant tissues [57]. It is worth to point out the involvement and co-expression of nitrate carriers, stress response and cell wall remodeling genes in unfavorable environments [23,58,59]. This complex co-expression network contributes to the tolerance under adverse conditions, allowing a quick allocation of resources from soil to shoots, even in the presence of salinity constraints. Such a mechanism would enhance nitrate assimilation upon drought, reorganize the root architecture in conditions of nutrient starvation and avoid the assimilation of toxic substances [60,61,62,63]. 

The vacuolar Fe transporter gi_351590806_gb_JP449264.1 was annotated as an ortholog of *At1g21140*; this gene encodes a nodulin-like 1 protein, whose transcript abundance is related to Fe deprivation [64]. This gene was reported to be part of a complex protein network able to regulate Fe acquisition and homeostasis under the control of ethylene and nitric oxide [65]. Moreover, three different LRR kinase receptors have been identified as downregulated by drought and related to N metabolism. This class of receptors is involved in stress response and in N fixing during root nodule symbiosis [66,67]. Particularly, these three hemp genes are orthologous to *At1g67720*, *At4g06744* and *At5g48740*. Using the eFP *Arabidopsis* browser [68], *At1g67720* and *At5g48740* showed an increased expression upon salinity, drought, heat, and oxidative stresses (not shown). Accordingly, genome-wide association studies (GWAS) on 1479 *Oryza sativa* accessions identified that 7.8% of the rice genome was improved by breeding. Among these, genes related to high affinity nitrate and ammonium transport showed a key role in improving rice varieties [69]. A similar approach could be transferred even to hemp breeding to obtain both high yielding and N starvation-tolerant varieties by regulating the expression of nitrate transporters.

Genes involved in the biosynthesis of secondary metabolites were identified in both up- and downregulated datasets under drought. For example, vinorine synthase was positively regulated by drought, while BAHD acyltransferase and salutaridinol 7-*O*-acetyltransferase were downregulated [24]. In addition, the KEGG category ko00910 (“*nitrogen metabolism*”) was significantly enriched upon water scarcity [24]. All the genes of this category showed a downregulation of the expression, thus highlighting a negative regulation of N metabolism upon drought stress.

Similarly, salt stress induced a complex transcriptional reorganization in two different hemp cultivars (Yunma 5 and Bamahuoma), by changing 220 upregulated and 249 downregulated genes in both genotypes [9]. We identified two upregulated and one downregulated N-related gene(s) influenced by salinity using the KEGG category “*nitrogen metabolism*” (ko00910). These three genes are the upregulated PK00197.1 (encoding a glutamate dehydrogenase isoform), PK06425.1 (encoding glutamate synthase) and the downregulated carbonic anhydrase (PK21222.1).

Glutamate dehydrogenase (GDH) and glutamate synthase (GOGAT) are crucial enzymes involved in N assimilation in plants. GOGAT is involved in basal and primary ammonium assimilation cycle, involving glutamine synthetase (GS/GOGAT cycle). On the other hand, the central role of GDH in balancing the flux of nitrogen compounds, particularly glutamate, cannot being ignored.

Interestingly, GDH operates by linking abiotic stresses such as drought, salinity, and heavy metal poisoning with nutrient starvation [70]. This association has been reported as a connection between metabolic adaptations and the protection of plants against ammonium accumulation; the presence of a molecular regulation of specific GDH isoenzymes induced by abiotic stresses and carbohydrate starvation was suggested [71]. These genes showed a significant critical role upon N starvation, inducing an increase in expression of GDH and GS in durum wheat [11]. Transgenic GS and GDH plants show a better regulation of N utilization, indicating that these candidate genes could be used for marker-assisted breeding strategies [72].

## 5. Breeding Strategies in Hemp: Nutrient Management and Synthesis of Secondary Metabolites

The renewed interest in hemp cultivation encouraged efforts in hemp breeding programs in the last 15 years [73,74]. These programs were finalized to obtain a better fiber quality, an increased yield andsynthesis of secondary metabolites, as well as to improve and control the time of flowering [75]. Breeding programs were performed by using various strategies, such as mass selection, cross, and hybrid breeding and using advanced technologies such as next-generation sequencing (NGS), genotype by sequencing (GBS), use for genetic maps [73,75,76]. Furthermore, breeding strategies and polyploidization induction also improve the production of secondary metabolites [77]. Large-scale rearrangements or duplications of genome enable new allelic combinations, by increasing the genetic variety and conferring physiological advantages in particular lineages [77]. The manipulation of ploidy status is a valuable and recognized tool for plant breeding that can induce the development of larger organs, by increasing heterozygosity, hybrid vigor; furthermore, this strategy can often be linked to an increased tolerance to stress [78]. A number of studies were recently reported about polyploidization in hemp [79,80,81], which naturally occurs as a diploid plant (2n = 20) [8]. Recently, the induction of polyploidy in *C. sativa* caused reduced CO_2_ fixation and carbohydrate content; an increase in flavonoid levels, possibly related to UV defense; reduction in cellulose content [79]. This was accompanied by an enhanced ability in nutrient uptake, thus highlighting an increased tolerance against starvation [79]. Mixoploid vegetative plants showed a higher content of Δ^9^-tetrahydrocannabinol (THC) and cannabidiol (CBD) [79].

N and P were shown to be essential in species with large genomes [82]. A study on 96-hexaploid wheat accessions reported an increased ability of N uptake in a polyploid population, suggesting this as an effective strategy to identify favorable QTLs for marker-assisted breeding programs [83].

It is worth noting that nutrient uptake and transport are key aspects in hemp, regulating the transition from vegetative to reproductive stage. The genes modulating this transition are thus interesting candidates for breeding to improve bast fiber quality [84].

The possibility to grow hemp in adverse environments is of high interest for breeding [75,85]. Specific cultivars of hemp have been selected for cultivation in specific countries and/or environments (e.g., Italy, France, Russia, China, USA), showing specific adaptations to low temperatures, drought, nutrient starvation and salinity [75]; furthermore, varieties showing phytoremediation potential have been obtained [86]. The relationship between nutrient availability and marketable products of hemp is a major topic for hemp breeding; particularly, the effects of N on hemp fibers are complex. N availability could influence hemp growth, plant harvesting, biomass production and fiber yield by impacting primary metabolism and the provision of precursors needed for cell wall synthesis [87].

## 6. Manipulation of the Cannabinoid Biosynthetic Pathway and Their Relation with N

Secondary metabolites, such as phytocannabinoids have application in the pharma industry, as well as cosmetics and the formulation of insecticides and antibacterial products [3,5,7,88,89,90]. Phytocannabinoids, such as THC and CBD, are secondary metabolites produced by hemp. THC is responsible for the psychoactive properties of hemp, whereas CBD is used against pain, anxiety, depression, and sleep disorders [6,91]. THC, cannabinol (CBN), cannabigerol (CBG) and CBD levels were studied in presence of N, P and potassium (K) supplementation in hemp cultivars grown for medical applications [12]. Interestingly, the concomitant N, P, and K supplementation showed physiological changes and modifications in the phytocannabinoid content. Fan leaf biomass increased with NPK and the addition of these elements also induced an increase in flower and stem biomass. These changes were accompanied by modified phytocannabinoid contents. THC levels decreased by 19% in inflorescence leaves of plants treated with NPK, while CBG increased by 71% in flowers. CBN levels decreased in flowers and inflorescence leaves by 38% and 36%, respectively, in the presence of NPK [12].

The major cannabinoids THC and CBD are both synthesized as acid forms from a common precursor, cannabigerolic acid (CBGA), by two different enzymes: THCA synthase (THCAS) and CBDA synthase (CBDAS) [92,93]. It has been proposed that THCAS and CBDAS are closely linked to each other, so that one of the two can be inactivated in drug- or hemp producing strains, respectively [80]. Tetraploid *Cannabis* strains producing enhanced THC and TCH/CDB levels showed phenotypes with larger leaves, increased density of trichomes and stomata and a rearrangement of secondary metabolites’ profile [81].

Recently, the enzymes responsible for the synthesis of cannabinoids (tetrahydrocannabinolic acid synthase and cannabidiolic acid synthase) were found to be secreted in trichomes’ exudates which is rich in hydrophilic, amphiphilic and osmoprotective metabolites providing the right micro-environment for the enzyme solubility and catalytic activity [94].

The first enzymes of the cannabinoid biosynthetic pathways were described and characterized [95,96]. Similarly, fatty acids and isoprenoids were originally identified as cannabinoid precursors [97]; particularly, hexanoyl-CoA and malonyl-CoA were recognized as critical compounds in the cannabinoid pathway. Using a combination of transcriptomic and mass spectrometry approaches in flowers from female hemp, node enzymes for the initial steps of cannabinoid biosynthetic pathway were identified. The enzyme responsible for the aldolic condensation between hexanoyl-CoA with three molecules of malonyl-CoA was identified by Gagne et al. [95]. This is the olivetolic acid cyclase (OAC), which catalyzes a C2–C7 intramolecular aldol condensation with carboxylate retention to form olivetolic acid. Similarly, *Cs*AAE1 and *Cs*AAE2 (acyl activating enzymes) were identified as major suppliers of hexanoyl-CoA [96].

These results were recently used to reproduce a complete cannabinoid biosynthetic route in yeast. This engineered pathway was reconstructed in *Saccharomyces cerevisiae* from galactose, by triggering a flux from the mevalonate pathway to geranyl pyrophosphate and by overexpressing olivetolic acid cyclase together with a geranylpyrophosphate:olivetolate geranyltransferase [98].

## 7. Conclusions

In this review, we illustrated how abiotic stresses and N metabolism are strictly interdependent in *C. sativa.* The relationship between nutrient availability and marketable products of hemp is a major topic for hemp breeding; in particular, N availability could influence hemp growth, plant harvesting, biomass production and fiber yield.

The results discussed strongly support the idea of a co-regulation of nitrate transporters, N metabolism-related genes, transcription factors and genes involved in secondary metabolism as part of a complex machinery engaged by hemp to counteract abiotic stress. Therefore, these gene families could represent potential targets for genetic improvement in hemp, as well as other fiber crops, ameliorating abiotic stress tolerance, nitrogen assimilation and, ultimately, production of biomass and secondary metabolites.

## Figures and Tables

**Table 1:** N resorption efficiency (NRE) and P resorption efficiency (PRE) of *C. sativa*, *A. scoparia*, *C. acuminatum*, and *P. communis* grown upon control (Ct), N addition (20 g of N/m^2^/year), P addition (4.4 g of P/ m^2^/year), and N + P conditions. Data taken from [28].

Plant	Control		N Supply		P Supply		N + P Addition	
	NRE (%)	PRE (%)	NRE (%)	PRE (%)	NRE (%)	PRE (%)	NRE (%)	PRE (%)
*C. sativa*	62	42	35	35	52	38	25	41
*P. communis*	42	10	45	20	58	10	60	15
*A. scoparia*	42	50	20	62	55	40	22	50
*C. acuminatum*	48	45	30	60	40	42	18	58

**Table 2:** List of genes related to N metabolism showing statistically-significant differences in expression (FDR-corrected *p*-value < 0.01) under drought- and salt-stress in *C. sativa*

Locus	Behavior vs. Abiotic Stress	Annotation	GO or KEGG Categories Related to N Metabolism
gi_351617093_gb_JP471394.1	UP upon Drought	*MYBR domain class transcription factor*	GO:0010243
gi_351606916_gb_JP461241.1	UP upon Drought	*WRKY transcription factor 33-like*	GO:0010243
gi_351617961_gb_JP472262.1	UP upon Drought	*Vinorine synthase-like*	GO:0006807
gi_351624360_gb_JP478661.1	UP upon Drought	*Triacylglycerol lipase*	GO:0006807
gi_351627864_gb_JP480747.1	UP upon Drought	*Vinorine synthase-like*	GO:0034641
gi_351612890_gb_JP467191.1	UP upon Drought	*Transcription factor LHY*	GO:0010243
gi_351603990_gb_JP458344.1	UP upon Drought	*Cysteine-rich receptor-like protein kinase*	GO:0071705
gi_351624708_gb_JP479009.1	UP upon Drought	*Delta-1-pyrroline-5-carboxylate synthetase*	GO:0034641
gi_351618788_gb_JP473089.1	UP upon Drought	*Pleiotropic drug resistance protein*	GO:0006995
gi_351599092_gb_JP453596.1	UP upon Drought	*ACD1-like*	GO:0044270
gi_351596616_gb_JP451172.1	UP upon Drought	*Probable peptide/nitrate transporter*	GO:0006807
gi_351625252_gb_JP479553.1	UP upon Drought	*Glucose-methanol-choline oxidoreductase*	GO:0006807
gi_351629105_gb_JP481988.1	UP upon Drought	*Hypothetical protein*	GO:0034641
gi_351628557_gb_JP481440.1	UP upon Drought	*Deoxytaxol N-benzoyltransferase*	GO:0006807
gi_351622676_gb_JP476977.1	UP upon Drought	*Shikimate O-hydroxycinnamoyltransferase*	GO:0034641
gi_351597997_gb_JP452531.1	UP upon Drought	*Aldehyde dehydrogenase*	GO:0006807
gi_351624288_gb_JP478589.1	UP upon Drought	*Hypothetical protein*	GO:0051171
gi_351615767_gb_JP470068.1	UP upon Drought	*MYB domain protein 20*	GO:0071705
gi_351623654_gb_JP477955.1	DOWN upon Drought	*21 kDa protein*	GO:0071705
gi_351629055_gb_JP481938.1	DOWN upon Drought	*Urea-proton symporter*	GO:0006995
gi_351624448_gb_JP478749.1	DOWN upon Drought	*Allantoinase*	GO:0006995
gi_351624658_gb_JP478959.1	DOWN upon Drought	*Salutaridinol 7-O-acetyltransferase*	GO:0006807
gi_351597746_gb_JP452287.1	DOWN upon Drought	*Anthranilate N-benzoyltransferase*	GO:0006807
gi_351605867_gb_JP460208.1	DOWN upon Drought	*Nitrate transporter 1.2*	GO:0006807
gi_351602034_gb_JP456469.1	DOWN upon Drought	*Nitrate transporter 1.2*	GO:0006807
gi_351619289_gb_JP473590.1	DOWN upon Drought	*Non-symbiotic hemoglobin 2*	GO:0009399
gi_351591331_gb_JP449779.1	DOWN upon Drought	*Protein PHR1-LIKE 1*	GO:0034641
gi_351625347_gb_JP479648.1	DOWN upon Drought	*Pleiotropic drug resistance protein 2*	GO:0006995
gi_351590806_gb_JP449264.1	DOWN upon Drought	*Vacuolar iron transporter*	GO:0009399
gi_351598838_gb_JP453346.1	DOWN upon Drought	*Pleiotropic drug resistance protein*	GO:0006995
gi_351620563_gb_JP474864.1	DOWN upon Drought	*Leucine-rich repeat family protein*	GO:0006995
gi_351597483_gb_JP452028.1	DOWN upon Drought	*LRR receptor-like serine/threonine kinase*	GO:0071705
gi_351596259_gb_JP450816.1	DOWN upon Drought	*Glutamine synthetase leaf isozyme*	GO:0009399/Ko00910
gi_351624862_gb_JP479163.1	DOWN upon Drought	*LRR receptor-like serine/threonine kinase*	GO:0071705
gi_351624507_gb_JP478808.1	DOWN upon Drought	*L-3-cyanoalanine synthase 1*	GO:0051410
gi_351623596_gb_JP477897.1	DOWN upon Drought	*Tropinone reductase*	GO:0044271
gi_351605608_gb_JP459952.1	DOWN upon Drought	*Major facilitator superfamily protein*	GO:0006807
gi_351606133_gb_JP460466.1	DOWN upon Drought	*BAHD acyltransferase*	GO:0006807
gi_351623568_gb_JP477869.1	DOWN upon Drought	*Tocopherol O-methyltransferase*	GO:0034641
gi_351601188_gb_JP455639.1	DOWN upon Drought	*LRR receptor-like serine/threonine kinase*	GO:0071705
gi_351598272_gb_JP452798.1	DOWN upon Drought	*Aminomethyltransferase, mitochondrial*	ko00910
gi_351612051_gb_JP466352.1	DOWN upon Drought	*Carbonic anhydrase 2*	ko00910
gi_351615730_gb_JP470031.1	DOWN upon Drought	*Carbonic anhydrase, chloroplastic*	ko00910
gi_351617853_gb_JP472154.1	DOWN upon Drought	*Carbonic anhydrase, chloroplastic*	ko00910
gi_351621906_gb_JP476207.1	DOWN upon Drought	*Bifunctional monodehydroascorbate reductase*	ko00910
PK00197.1	UP upon Salinity	*Glutamate dehydrogenase*	ko00910
PK06425.1	UP upon Salinity	*Glutamate synthase*	ko00910
PK21222.1	DOWN upon Salinity	*Carbonic anhydrase 1*	ko00910
